# A Rare Manifestation of a Rare Disease: Nodular Lymphocyte-Predominant Hodgkin Lymphoma Presenting As Paraneoplastic Aseptic Meningitis

**DOI:** 10.7759/cureus.86327

**Published:** 2025-06-18

**Authors:** Aishwarya Saripalli, Elaine Deemer, Kasey Fox

**Affiliations:** 1 Internal Medicine, University of California Los Angeles (UCLA) Kern Medical, Bakersfield, USA; 2 Geriatrics, University of California Los Angeles David Geffen School of Medicine, Los Angeles, USA

**Keywords:** chronic meningitis, nodular lp hodgkin lymphoma, paraneoplastic, presentation, staging, treatment

## Abstract

Nodular lymphocyte-predominant Hodgkin lymphoma (NLPHL) is an uncommon form of Hodgkin lymphoma (HL). It commonly presents as chronic, asymptomatic, slow-growing peripheral lymphadenopathy. Central nervous system involvement in NLPHL is rare.

Here we report a case of a 28-year-old male patient who presented with a seven-week history of headache and neck pain. The evaluation revealed a diagnosis of subacute meningitis. He was newly diagnosed with NLPHL on lymph node biopsy. With this presentation of subacute aseptic meningitis with a negative workup for infectious, autoimmune etiology, and absence of malignant cells in the CSF, in the setting of newly diagnosed NLPHL, a diagnosis of paraneoplastic aseptic meningitis was made. The patient improved symptomatically with the treatment of his cancer.

## Introduction

Hodgkin lymphoma (HL) is one of the most common cancers in adolescents and adults aged 15-39 years [1]. There are two main types of HL: the first is classic HL, which is further sub-typed as nodular sclerosis, lymphocyte-rich, mixed cellularity, and lymphocyte-depleted, and the second is nodular lymphocyte-predominant HL (NLPHL).

Central nervous system involvement in NLPHL is exceedingly rare, which classically presents as asymptomatic, slow-growing peripheral lymphadenopathy. Here we describe a unique case of paraneoplastic aseptic meningitis as the initial presentation of a patient with nodular lymphocyte-predominant Hodgkin lymphoma.

This case report was presented at the American College of Physicians National Conference 2025 and Western Medical Research Conference 2025.

## Case presentation

A 28-year-old male patient with a history of hypertension and obesity presented to the emergency department with complaints of headache and neck pain for the past seven weeks. His symptoms initially began as a bilateral headache with a band-like sensation, which over the subsequent one to two weeks progressed to include neck pain, with worsening severity. He did not endorse any fever, weight loss, weakness of extremities, seizures, or blurring of vision. The review of systems was otherwise negative. On evaluation, the patient had normal vital signs; he appeared to be visibly in distress and was noted to have bilateral papilledema on bedside exam. Examination of the nervous system was unremarkable, with no meningeal signs. The CT brain without contrast was unremarkable. Lumbar puncture was performed, which was remarkable for an opening pressure of 23 cm of H_2_O, WBC count of 1245, 95% lymphocytes, RBC count of 123, protein 148, and glucose of 63 (Table 1).

**Table 1:** CSF analysis results

Test result	Value with units	Reference range
Opening pressure	23 cm of H_2_O	7-15 cm of H_2_O
CSF total WBC count	1245 cells/ mm^3^	0-5 cells/ mm^3^
Differential WBC count	95% lymphocytes	-
RBC count	123 cells/ mm^3^	0-5 cells/ mm^3^
CSF protein	148 mg/dL	15-60 mg/dL
CSF glucose	63 mg/dL	50-80 mg/dL

A diagnosis of subacute meningitis was made, and the patient was admitted for further evaluation. MRI Brain and cervical spine with and without contrast was done and was unremarkable, with no parenchymal lesion or abnormal meningeal enhancement. Neurology and infectious disease were consulted to assist with further workup. He was started on empiric treatment for coccidioidomycosis and tuberculosis pending results of further diagnostic testing, including bacterial, viral, fungal, parasitic, and autoimmune etiologies, all of which resulted negative. CSF immunophenotyping revealed only mature T-cells. We reinterviewed the patient, and he noted having a small lump in his axilla that had been slowly growing for a few months. Examination revealed bilateral axillary lymphadenopathy, which was missed on initial exam, and no cervical or inguinal lymphadenopathy. Excisional lymph node biopsy was performed, which showed large “popcorn cells” with co-expression of CD20 and OCT2, with a lack of CD15 and CD30, which were consistent with NLPHL. With this presentation of subacute aseptic meningitis with negative workup for infectious, autoimmune etiology, and absence of malignant cells in the CSF, in the setting of newly diagnosed NLPHL, the patient was diagnosed with paraneoplastic aseptic meningitis. Oncology was consulted for definitive management; however, the patient opted to continue treatment in another city closer to home. On follow-up via telephone, he reported improvement in symptoms with cancer treatment.

## Discussion

NLPHL makes up approximately 5% of all HL cases [1]. In the United States and Europe, the incidence of NLPHL has remained stable at around eight to nine cases per 10 million people annually; however, rates in children may be increasing [2,3]. Approximately 75% of individuals diagnosed with NLPHL are male [2]. In the United States, NLPHL occurs more frequently among Black Americans than White Americans, a pattern that contrasts with classic HL [2].

The malignant cells of NLPHL (denoted as lymphocyte-predominant or LP cells) are derived from germinal center B (GCB) cells. Similar to normal germinal center B cells, the growth and survival of LP cells appear to rely on signaling through the immunoglobulin receptor, as well as interactions with dendritic cells and follicular T cells within B-cell follicles [4]. LP cells, also known as "popcorn cells," are large with a single, vesicular, multilobed nucleus and small, distinct nucleoli typically located at the nuclear periphery, without surrounding perinuclear halos [5]. The distinct immunophenotype of LP cells plays a key role in differentiating NLPHL from classic HL. LP cells express B-cell markers such as CD19, CD20, CD22, and CD79a, as well as CD45 and the transcription factors BOB-1 and OCT-2. They lack CD15 expression and only rarely express CD30 [1,6]. Because LP cells produce few proinflammatory cytokines, systemic B symptoms such as fever, night sweats, and weight loss are uncommon in NLPHL. Epstein-Barr virus (EBV) is infrequently detected in NLPHL. Rare cases of EBV-associated NLPHL often exhibit atypical features that resemble those of lymphocyte-rich classic HL [7].

NLPHL is characterised by an indolent course and occasional late relapse. It generally presents as chronic, asymptomatic peripheral lymphadenopathy. Involvement of bone marrow, mediastinal, liver, and spleen is infrequent. The majority of patients present with early-stage disease. CNS involvement in HL is exceedingly rare. One multi-center international retrospective analysis reported a prevalence of 0.07% in classic HL [8]. Similar data for NLPHL is unavailable, given that NLPHL by itself is a rare entity. Neurological manifestations of lymphomas can be direct (metastatic) or indirect. Direct complications include intraparenchymal, dural, leptomeningeal, or epidural spinal cord involvement. Indirect complications include paraneoplastic, granulomatous angiitis of the CNS, or treatment-related complications [9]. Paraneoplastic neurological syndromes result from a misguided immune response to the nervous system triggered by cross-reactivity between nervous system tissue and a distant tumor [10]. This antibody-mediated immune response triggers meningeal inflammation, resulting in an aseptic meningitis. The classically described paraneoplastic syndromes of the central nervous system are limbic encephalitis, encephalomyelitis, cerebellar degeneration, brainstem encephalitis, Opsoclonus-Myoclonus Syndrome, myelopathies, and Stiff Person Syndrome. However, paraneoplastic pathology can affect any part of the nervous system, producing a wide variety of clinical manifestations.

Staging of NLPHL, like classic HL, is based on clinical evaluation and PET/CT findings according to Lugano criteria and may be classified as favorable or unfavorable disease based on size of lymph nodes (> 10 cm), number of sites (> 3) involved and level of erythrocyte sedimentation rate (ESR) elevation (>50 mm/hour). Prognosis is determined by the International Prognostic Score (IPS), which includes laboratory values of albumin, hemoglobin, leukocyte and lymphocyte counts, patient characteristics like age and sex, and stage of the disease.

Primary treatment is based on staging, as described below. Stage IA or contiguous II A non-bulky disease is observation or involved site radiation therapy (ISRT). Bulky disease is defined as greater than 10 cm. Stage IB, IIB, or Stage IA, IIA with bulky disease is chemotherapy plus rituximab and ISRT. Stage II A with non-contiguous disease is chemotherapy plus rituximab with or without ISRT or single-agent rituximab. For stage III or IV disease, observation is appropriate if the patient is asymptomatic and/or based on clinical judgement. Other treatment options include chemotherapy plus rituximab, with or without ISRT, or single-agent rituximab or local radiation therapy for palliation of locally symptomatic disease. Chemotherapy regimens used in the above treatment include ABVD (doxorubicin, bleomycin, vinblastine, dacarbazine), CHOP (cyclophosphamide, doxorubicin, vincristine, prednisone), and CVbP (cyclophosphamide, vinblastine, prednisone). In patients who have contraindications to receiving anthracyclines, bendamustine plus rituximab is an alternate option.

After primary treatment, PET/CT is used to restage the disease as response, stable, or progressive disease. Patients categorized as stable or progressive disease on PET/CT are evaluated with repeat biopsy to look for positive or negative disease. If the patient is noted to have positive disease on biopsy, the NLPHL is considered refractory. Patients categorized as having a response or negative disease on biopsy are managed by observation/ surveillance. Surveillance is continued for five years after treatment and complete response.

Our patient presented with a thus far unreported clinical manifestation of NLPHL. This case highlights not only an uncommon presentation of this disease, but it also reiterates the importance of thorough history and detailed physical examination during evaluation of subacute or chronic meningitis. Although testing for onconeural antibodies could not be performed in our case, improvement of symptoms after cancer therapy suggested a paraneoplastic etiology.

## Conclusions

NLPHL can have atypical presentations such as neurological manifestations, including paraneoplastic syndromes. A thorough history and physical examination are key in evaluating chronic meningitis. Paraneoplastic syndromes are often a diagnosis of exclusion and have variable presentations. Improvement of symptoms with treatment of the underlying malignancy is often the most reliable confirmation of these diagnoses. Testing for onconeural antibodies in subsequent cases may help identify specific antibodies associated with paraneoplastic meningitis.
